# Global sensitivity analysis of roof hazard factors based on information entropy and the surrogate model

**DOI:** 10.1038/s41598-025-85988-y

**Published:** 2025-02-10

**Authors:** Guowei Zhang, Ting Ren, Jun Han

**Affiliations:** 1 College of Mining, Liaoning Technical University, Fuxin, 123000 China; 2 University of Wollongong, Wollongong, NSW 2522 Australia

**Keywords:** Co-occurrence matrix, Information entropy, Surrogate model, Sobol’s method, Hazard factors sensitivity analysis, Natural hazards, Engineering

## Abstract

The prevention and management of coal mine roof accidents remain challenging issues because it is difficult to evaluate and quantify the interaction effects of the disaster hazard factors objectively. This paper proposes a novel approach: combining information entropy and the surrogate model—and applies Sobol’s method, aiming to solve it and to obtain the hazard factors’ 1th and the global sensitivity value without human intervention. The results show that: (1) The complex logical relationships and interactions of roof hazard factors can be transformed into quantifiable numerical values by building a co-occurrence matrix of disaster factors and calculating its information entropy. (2) The sensitivity levels of roof hazard factors can be successfully distinguished and categorized into priority management and prevention or general management and prevention using the surrogate model and Sobol’s sensitivity method. The novel sensitivity analysis approach suggested in this study considers both the individual impacts of hazard factors and their interactions, offering a more thorough framework for risk assessment as well as a fresh perspective and tool for coal mine safety research.

## Introduction

Coal was, is, and will continue to be China’s primary energy source in the forthcoming years despite facing the pressure and challenge of environmental protection and energy transition, according to the reports^1,2^ from IEA and Energy Institute. As mining depth increases, the coal roadway’s plastic zone grows under high stress, with the more complex the geological conditions and the more broken the roof or locally top coal, causing the stability of the roadway supporting structure to deteriorate^3^, which gradually results in the top coal of the roadway to fall, bolts or anchor cables to break or fracture, and even causes the roof to collapse or leave a large area of the roof empty, and so on^4,5^. Moreover, coal mine roof accidents are frequently caused by several intricate factors and their interactions, and the complexity and uncertainty of these factors make accident prevention and management extremely difficult. Thus, it is crucial to conduct thorough research on the hazard factors and their sensitivity to coal mine roof accidents in order to raise the standard of coal mine safety management and prevent roof accidents.

Currently, many state-of-the-art research studies have been conducted in the field of coal mine surrounding rock control, which can reasonably provide prevention and control decision support for managers and project implementers and can be summarized into two categories. One is building a thorough analysis model or evaluation system to raise the degree of risk management and accident control for roofs, e.g., decision tree framework^6,7^, fault tree analysis^8^, Monte Carlo method^9^, Weibull and Poission distribution models^10^, time series theory analysis^11^, fuzzy comprehensive evaluation method^12,13^, Ontology-based semantic model^14^, improved AHP method^15^, Bayesian network^16,17^, triangular fuzzy theory^18^, mutation theory^19^, DEMATEL-ANP method^20^, complex network^21^. The other is using mathematical modeling to identify and analyze critical (potential) risk factors, such as exploring the typical hazard (risk) elements^21–26^.

Being one of the primary coal mine disasters, many people are gradually realizing that its nature is a complex system with discrete, random, and uncertain states that, when combined, make the accident’s causes intricate and unpredictable. After that, a tiny portion of people started to investigate risk coupling progressively after concentrating on the inner link between the hazard factors, e.g., superimposed risk^26^, interactive models^27–30^, the coupling effect^31,32^; as well as complex risk interaction systems in other coal mine accident fields^33,34^, which reinforces the necessity and urgency in this direction.

Given all the above, research can be summed up in two aspects: first, there are very few papers that reasonably consider the inner interaction between the hazard factors of roof accidents; second, risk superposition is only taken into consideration in the logical sense, which simplifies the issue but fails to fully account for the complexity of its interaction. As a result, it is challenging to accurately identify and prioritize control of the key hazard factors in actual security management.

To fill this gap, we offer a new methodology that fully considers the inner interaction among hazard factors and applies information entropy theory to the quantitative analysis of roof hazard factors for the first time. Additionally, the 1th and global sensitivity values of roof hazard factors are quantitatively analyzed by the surrogate models and Sobol’s method, revealing the impact level of each hazard factor and offering a more comprehensive and systematic framework for risk assessment. In addition to improving risk assessment accuracy, this approach can offer new analytical tools and theoretical backing for coal mine safety management. The goal of this paper is to give decision-makers a solid scientific foundation for preventing and controlling coal mine roof accidents.

## Theory and methodology

Assuming the sensitivity value of roof accident hazard factors is the ultimate outcome, it appears that we just need to put the input variable values through the known function mapping and then everything will be fine. The issue appears more complex, though, and the specifics are as follows: How can hazard factors be converted into digital numbers in a reasonable and objective manner? And how to use them to build a function mapping? Even how to handle and simplify the complex network relationships of their inner interaction effect, if they exist.

**Fig. 1:**
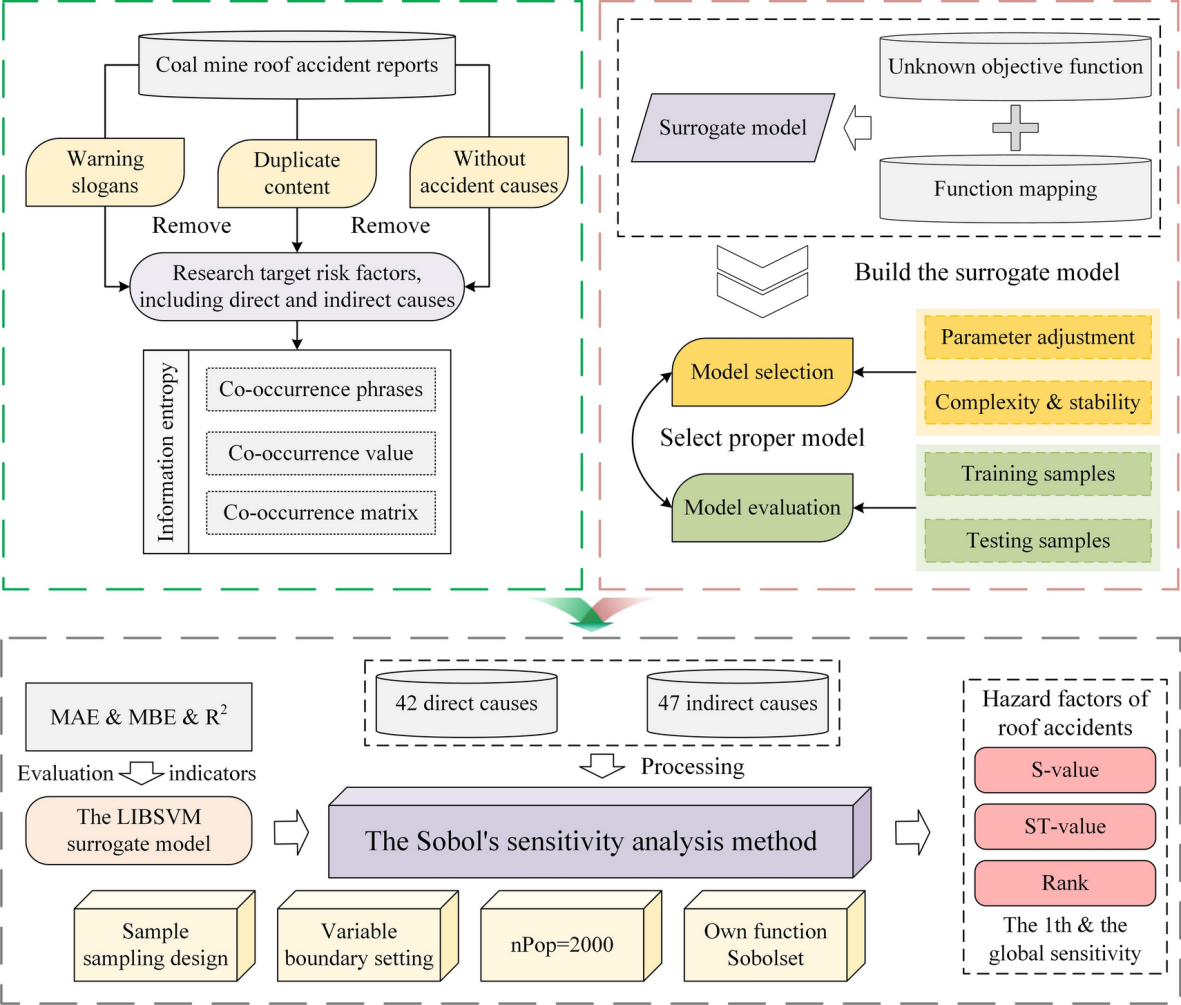
Flowchart of hazard factor sensitivity analysis.

To tackle this thorny problem, displayed in Fig. 1, firstly, transforming the hazard phrases that occurred simultaneously in the same accident report into the co-occurrence value, then together forming the co-occurrence matrix, achieved assigning the initial input values of hazard factors and considering their inner logical relationship. Moreover, intelligent algorithms with appropriate classification prediction performance can be used to obtain the function mapping relationship. The algorithm can mimic and perceive the potential relationship by using some of the factors as training samples and the remaining ones as testing samples. Finally, integrate Sobol’s method—distinctive mathematical ideas and concepts—into a calculation procedure that can resolve factor interaction effects using single or multiple effect functions.

### Principle of co-occurrence matrix

1. Co-occurrence phrases. This is an assumption, i.e., hazard factors that appear simultaneously in the roof accident report of a certain coal mine. For example, the four hazard factors A, B, C, and D can be used to replace the cause of a roof accident; this is known as the co-occurrence phrases of the roof accident.
2. Co-occurrence value. This indicates the number of times different hazard factors have been shown simultaneously in a single accident report. This means that if one cause of a roof accident can be simplified to [A, B, A, C, D, E, and C], then these B, C, D, and E are the co-occurrence phrases of hazard factor A. The co-occurrence values of A and B, A and C are 2 and 4, respectively, and the rest can be obtained in the same manner.
3. Co-occurrence matrix^35^. The co-occurrence values of all the coal mine roof accident hazard factors are represented by this matrix, which can be used to deeply analyze the hidden relationships between them and reveal the ontology-based semantic logic and connections.

A co-occurrence matrix containing *m* hazard factors is defined to be^36,37^:

1 $$CM = \left[ {\begin{array}{*{20}l} {c_{11} } \hfill &\quad {c_{12} } \hfill &\quad \ldots \hfill &\quad {c_{1m} } \hfill \\ {c_{21} } \hfill &\quad {c_{22} } \hfill &\quad \ldots \hfill &\quad {c_{2m} } \hfill \\ \vdots \hfill &\quad \vdots \hfill &\quad \ddots \hfill &\quad \vdots \hfill \\ {c_{m1} } \hfill &\quad {c_{m2} } \hfill &\quad \ldots \hfill &\quad {c_{mm} } \hfill \\ \end{array} } \right]$$

where *c*_*ij*_ is the co-occurrence values of *i* and *j*; and with *i* = *j*, we have *c*_*ij*_ = 0, while $i \ne j$, the *c*_*ij*_ is the concrete value.

Converted to an upper triangular symmetric matrix in order to easily count and calculate, and the row elements *i* in the order while the column elements *j* in the reverse order, then Eq. (1) can be simplified as:

2 $$CM = \left[ {\begin{array}{*{20}l} {c_{1m} } \hfill &\quad \ldots \hfill &\quad {c_{12} } \hfill &\quad 0 \hfill \\ {c_{2m} } \hfill &\quad \ldots \hfill &\quad 0 \hfill &\quad {c_{21} } \hfill \\ \vdots \hfill &\quad \vdots \hfill &\quad \ddots \hfill &\quad \vdots \hfill \\ 0 \hfill &\quad \ldots \hfill &\quad {c_{m2} } \hfill &\quad {c_{m1} } \hfill \\ \end{array} } \right]$$

With a large value of *c*_*ij*_ and with $i \ne j$, the hazard factors *i* and *j* have a high frequency in the same text of the roof accident reports, indicating that they are more closely related to each other^36,37^.

### Theory of information entropy

No one would deny that entropy is a critical concept in physics and information theory. In physics, entropy is a quantity that measures the degree of disorder in a system, which is related to the second law of thermodynamics; while in information theory, Claude Shannon first introduced entropy, also known as Shannon’s entropy, as an uncertainty or the average amount that measures information in a message^38,39^.

The larger the information entropy, the greater the uncertainty of information, or the greater the average amount of information that has been received^39,40^. The corresponding relationships can be written as follows.

If any discrete or continuous variables *x* satisfy the probability distribution *p*(*x*), the formula is expressed, respectively, as:

3 $$Entropy = \sum\limits_{x \in X} {p\left( x \right)\log p\left( x \right)}$$

4 $$Entropy = - \int {p\left( x \right)\log p\left( x \right)} dx$$

where *x* ~ *P*(*x*), denotes the calculated expectation by the probability distribution *p*(*x*), it also can be simplified as:

5 $$E\left( X \right) = E_{x\sim p(x)} \left[ { - \log p\left( x \right)} \right]$$

### Definition of surrogate model

The surrogate model, also known as the response surface model or meta-model simulator, is a simplified model used to progress the issue of approximately complex or time-consuming calculations^41,42^. However, we had to create an optimization mechanism that employs a reasonable surrogate model strategy to approximate and obtain the global optimal solution because it is hard to accurately obtain the corresponding objective functions for some questions. This ensures that the results sequence will accurately converge to the problem’s actual solution. The model is simplified and written as follows.

Assuming an optimization problem of a complex system with *m* factors, and there is an approximate model between the factors $x = [x_{1} ,x_{2} , \ldots ,x_{m} ]^{T} \in R^{m}$ and the unknown objective function $y:R^{m} \to R$ (also called the constraint function)^42^. The first step is to construct *n*-dimensional sample points in *R*-space.

6 $$X = \left[ {x^{(1)} ,x^{(2)} , \ldots ,x^{(m)} } \right]^{T} \in R^{n \times m}$$

Then, obtain the corresponding function mapping relationship by computing numerically the *n*-dimensional sample points:

7 $$Y = \left[ {y\left( {x^{(1)} } \right),y\left( {x^{(2)} } \right), \ldots ,y\left( {x^{m} } \right)} \right]^{T} \in R^{m}$$

A many-to-one mapping between these sample points and the function mapping relationship creates a sample dataset $S(x,y(x))$.

Moreover, we need pay attention to the following matters:

1. Model selection. Which surrogate model would be selected? It is dependent on the nature of the issue and the level of accuracy needed, as well as other factors such as the difficulty of parameter adjustment, the model’s complexity, and stability, etc.
2. Data training. A surrogate model usually needs a certain amount of data to train the initial parameters, or random initial thresholds. The model’s accuracy is directly impacted by the selection and quality of training data.
3. Variable boundary. To ensure the validity and accuracy of the surrogate model, the corresponding variables’ upper and lower boundaries should be set and unable to exceed the upper and lower limits of the research data.

### Method of sensitivity analysis

It is common to encounter some complex problems, e.g., non-linear problems, problems lacking an objective function, and problems where it is difficult to clarify how the common interaction of several input variables will affect the results.

However, as we know, Sobol’s sensitivity analysis method considers that the total variance is superimposed by two components, with one generated by the individual variables and the other generated by the interactions between the variables, and the core concepts are as follows^43–45^.

Assuming the space *R*^*n*^ = {*z*|*x*_*i*_ ∈ [0, 1]; *i* = 0, 1, …, *m*} and function mapping *f*(*x*) composed by the *n*-dimensional independent variable with *m* input variables, then decomposed *y* = *f*(*x*) into a combination of a single effect function and multiple effect functions. The general expressions are given as follows:

8 $$f\left( x \right) = f_{0} + \sum\limits_{k = 1}^{m} {\sum\limits_{{i_{1} < \cdots < i_{k} }}^{m} {f_{{i_{1} , \ldots i_{k} }} \left( {x_{{i_{1} }} ,x_{{i_{2} }} , \ldots ,x_{{i_{k} }} } \right)} }$$

9 $$s.t.\int {f_{{i_{1} ,i_{2} , \ldots i_{k} }} } \left( {x_{{i_{1} }} ,x_{{i_{2} }} , \ldots ,x_{{i_{k} }} } \right)dx_{{i_{s} }} = 0,\;\;1 \le s \le k$$

where *f*_0_ is a constant, the condition satisfied by the function relation (8) can be written as (9). The input variables are *x*_*i*_, *x*_*j*_, and *x*_*m*_, and their total number is *m*.

Moreover, *f*_*i*_(*x*_*i*_), *f*_*i*,*j*_(*x*_*i*_, *x*_*j*_), and *f*_1,2,…*m*_(*x*_1_, *x*_2_, …, *x*_*m*_) represent single, double, and *m* effect functions, respectively, with one, two, and *m* variables. If *m* is the total number of variables, then *f*_1,2,…*m*_(*x*_1_, *x*_2_, …, *x*_*m*_) is also referred to as the total effect function. Due to the objective function mapping model *f*(*x*)’s one-variable effects, the variance *V*_*i*_ is as follows^43–45^:

10 $$V_{i} = \int {f_{i}^{2} \left( {x_{i} } \right)dx_{i} }$$

The partial variance $V_{{i_{1} ,i_{2} , \ldots i_{k} }}$ resulting from the interaction effect between the parameters is denoted as:

11 $$V_{{i_{1} ,i_{2} , \ldots i_{k} }} = \prod \int {f_{{i_{1} ,i_{2} , \ldots i_{k} }}^{2} } \left( {x_{{i_{1} }} ,x_{{i_{2} }} , \ldots ,x_{{i_{k} }} } \right)dx_{{i_{1} }} \ldots dx_{{i_{k} }}$$

While the total variance *V* resulting from the interaction effect of all input variables is expressed as:

12 $$V = \sum\limits_{k = 1}^{m} {\sum\limits_{{i_{1} < \cdots < i_{k} }}^{m} {f_{{i_{1} \ldots i_{k} }}^{2} \left( {x_{{i_{1} }} ,x_{{i_{2} }} , \ldots ,x_{{i_{k} }} } \right)} } dx_{{i_{1} }} \ldots dx_{{i_{k} }}$$

Assumption: the variable$X_{i}$ (*i* = 1, 2, …, *m*) is independently and identically distributed in the objective function, the conditional variance of *Y* is *Var*(*Y*|*X*_*i*_) when *X*_*i*_ = *x*_*i*_, and the difference between *Var*(*Y*) and *Var*(*Y*|*X*_*i*_) illustrates how *X*_*i*_ affects *Y*^45^.

With *Var*(*Y*) = $E_{{X_{i} }} \left( {Var_{{X_{\sim i} }} \left( {Y|X_{i} } \right)} \right) + Var_{{X_{i} }} \left( {E_{{X_{\sim i} }} \left( {Y|X_{i} } \right)} \right)$, and when variable interaction effects are disregarded or considered, the variable sensitivity is as follows, respectively:

13 $$S_{{X_{i} }} = \frac{{Var_{{X_{i} }} \left( {E_{{X_{\sim i} }} \left( {Y|X_{i} } \right)} \right)}}{Var\left( Y \right)}$$

14 $$S_{{X_{i} }}^{T} = \frac{{Var\left( Y \right) - Var_{{X_{\sim i} }} \left( {E_{{X_{i} }} \left( {Y|X_{\sim i} } \right)} \right)}}{Var\left( Y \right)}$$

Considering $Var(Y) = E_{{X_{i} }} \left( {V_{{X_{\sim i} }} \left( {Y|X_{i} } \right)} \right) + V_{{X_{i} }} \left( {E_{{X_{\sim i} }} \left( {Y|X_{i} } \right)} \right)$, Eq. (14) can be simplified as:

15 $$S_{{X_{i} }}^{T} = \frac{{E_{{X_{\sim i} }} \left( {Var_{{X_{i} }} \left( {Y|X_{\sim i} } \right)} \right)}}{Var\left( Y \right)}$$

where the 1th sensitivity of *X*_*i*_ is $S_{{X_{i} }}$, indicating the contribution of one-variable *X*_*i*_ to the variance of *Y* and meeting $S_{{X_{i} }} \in \left[ {0,1} \right]$; while $Var_{{X_{\sim i} }} \left( {E_{{X_{i} }} \left( {Y|X_{\sim i} } \right)} \right)$ denotes the contribution to the variance of *Y* for all variables except variable *X*_*i*_, and its opposite event is $Var\left( Y \right) - Var_{{X_{\sim i} }} \left( {E_{{X_{i} }} \left( {Y|X_{\sim i} } \right)} \right)$, which is the contribution to *Y* for all variables that have an interaction effect. Then, the global sensitivity of *X*_*i*_ is $S_{{X_{i} }}^{T}$ ($S_{{X_{i} }}^{T} \in \left[ {0,1} \right]$), which contains the interaction-effect relationship among all variables.

It is evident from relations (13)–(15) that the larger values of $S_{{X_{i} }}$ and $S_{{X_{i} }}^{T}$ mean that the input variable *X*_*i*_ (single or not) has a greater effect on the output variance, respectively, suggesting that the input variable is more sensitive to the results. If the values are smaller, indicating that not only does the input variable itself have a small effect on the result, but also that the interaction effect between the input is small.

### Algorithm design for sample sampling

As a matter of fact, for the unknown objective function, it is necessary to generate certain sequences of sample sampling in order to ensure the convergence of the model, the richness of the samples, and the accuracy of the results, which is usually done by Monte Carlo methods^46^, Latin Hypercube Sampling^47,48^, and Orthogonal Design^49^, etc. The principle is to generate a matrix of multiple groups by sample sampling:

16 $$A = \left[ {\begin{array}{*{20}l} {x_{11} } \hfill & {\quad x_{12} } \hfill & {\quad \ldots } \hfill & {\quad x_{1m} } \hfill \\ {x_{21} } \hfill & {\quad x_{22} } \hfill & {\quad \ldots } \hfill & {\quad x_{2m} } \hfill \\ \vdots \hfill & {\quad \vdots } \hfill & {\quad \ddots } \hfill & {\quad \vdots } \hfill \\ {x_{n1} } \hfill & {\quad x_{n2} } \hfill & {\quad \ldots } \hfill & {\quad x_{nm} } \hfill \\ \end{array} } \right]$$

17 $$B = \left[ {\begin{array}{*{20}l} {x_{11}^{\prime } } \hfill & {\quad x_{12}^{\prime } } \hfill & {\quad \ldots } \hfill & {\quad x_{1m}^{\prime } } \hfill \\ {x_{21}^{\prime } } \hfill & {\quad x_{22}^{\prime } } \hfill & {\quad \ldots } \hfill & {\quad x_{2m}^{\prime } } \hfill \\ \vdots \hfill & {\quad \vdots } \hfill & {\quad \ddots } \hfill & {\quad \vdots } \hfill \\ {x_{n1}^{\prime } } \hfill & {\quad x_{n2}^{\prime } } \hfill & {\quad \ldots } \hfill & {\quad x_{nm}^{\prime } } \hfill \\ \end{array} } \right]$$

As shown in Eq. (18), create matrix *AB*_*k*_ by swapping out the *k*th column of matrix *A* for the *k*th column of matrix *B*.

18 $$AB_{k} = \left[ {\begin{array}{*{20}l} {x_{11} } \hfill & {\quad x_{12} } \hfill & {\quad \ldots } \hfill & {\quad x_{1k}^{\prime } } \hfill & {\quad \ldots } \hfill & {\quad x_{1m} } \hfill \\ {x_{21} } \hfill & {\quad x_{22} } \hfill & {\quad \ldots } \hfill & {\quad x_{2k}^{\prime } } \hfill & {\quad \ldots } \hfill & {\quad x_{2m} } \hfill \\ \vdots \hfill & {\quad \vdots } \hfill & {\quad \ddots } \hfill & {\quad \vdots } \hfill & {\quad \ddots } \hfill & {\quad \vdots } \hfill \\ {x_{n1} } \hfill & {\quad x_{n2} } \hfill & {\quad \ldots } \hfill & {\quad x_{nk}^{\prime } } \hfill & {\quad \ldots } \hfill & {\quad x_{nm} } \hfill \\ \end{array} } \right]$$

After constructing the matrix *AB*_*k*_, 2*AB*_*k*_ is obtained as the initial input, and the multiple effect function of the variable is^46^:

19 $$Var_{{X_{i} }} \left( {E_{{X_{\sim i} }} \left( {Y|X_{i} } \right)} \right) \approx \frac{1}{N}\sum\limits_{j = 1}^{M} {f\left( B \right)_{j} \left( {f\left( {AB^{i} } \right)_{j} - f\left( A \right)_{j} } \right) }$$

20 $$E_{{X_{\sim i} }} \left( {Var_{{X_{i} }} \left( {Y|X_{\sim i} } \right)} \right) \approx \frac{1}{2N}\sum\limits_{j = 1}^{M} {\left( {f\left( A \right)_{j} - f\left( {AB^{i} } \right)_{j} } \right)^{2} }$$

After choosing a proper surrogate model, the number of sampling samples needs to be set reasonably according to the model’s convergence, followed by setting the quantity of input variables, which is equal to the matrix’s column dimension, normalizing the initial input values, i.e., data standardization, and setting upper and lower boundaries, and then outputting the 1th and the global sensitivity values.

## Research and analysis

### Explanation about research data

This paper, which is based on the concept of big data analysis, focuses on the text portion of the direct and indirect causes in the coal mine roof accident reports that were published by the State Coal Mine Safety Supervision Bureau, Coal Mine Safety Website, Safety Management Website, and so on. The research object covered two significant periods of China’s 13th Five-Year Plan and supply-side structural reform as well as the early stages of the 14th Five-Year Plan. Then, 393 coal mine roof accident reports were collected and taken as the initial data source by web crawlers.

After eliminating textual data types like “coal mine warning slogans”, “duplicate content”, and reports without “direct causes and indirect causes”, 115 roof accident reports with detailed direct and indirect causes were cherry-picked and gathered as the standard research objects of hazard factors analysis, which covers the period from January 1, 2016, to December 31, 2023. After using optical character recognition technology to convert these standard accident reports into text format of UTF-8 or GBK, we were able to obtain hazard factors, including 42 direct causes and 47 indirect causes, after using Hanzi Regular and Chinese word segmentation to cut them into individual words (phrases) that represent the key information details of the roof accidents’ causes and to retain high-frequency occurrences that correspond with the accidents.

### Preprocessing: parameter and model debugging

#### Information theory calculation

Calculate the information entropy of hazard factors based on the co-occurrence matrix of the direct and indirect causes of roof accident reports. The minus sign in formulae (3)–(5) is to ensure that the final value is positive or zero, avoiding a negative result^39^. Moreover, base-a logarithmic function only needs to satisfy that a low probability event *x*_*i*_ corresponds to a high information content and is usually chosen to satisfy the requirements of *a* ≥ 2, *a* ∈ *R*. For example, in a roof accident report, there are combinations, including three times A, B, and C hazard factors and two times D, B, and E. The left-information entropy of the hazard factor B is:

$$E\left( {B_{left} } \right) = - \frac{3}{5}\log \left( \frac{3}{5} \right) - \frac{2}{5}\log \left( \frac{2}{5} \right)$$

**Fig. 2:**
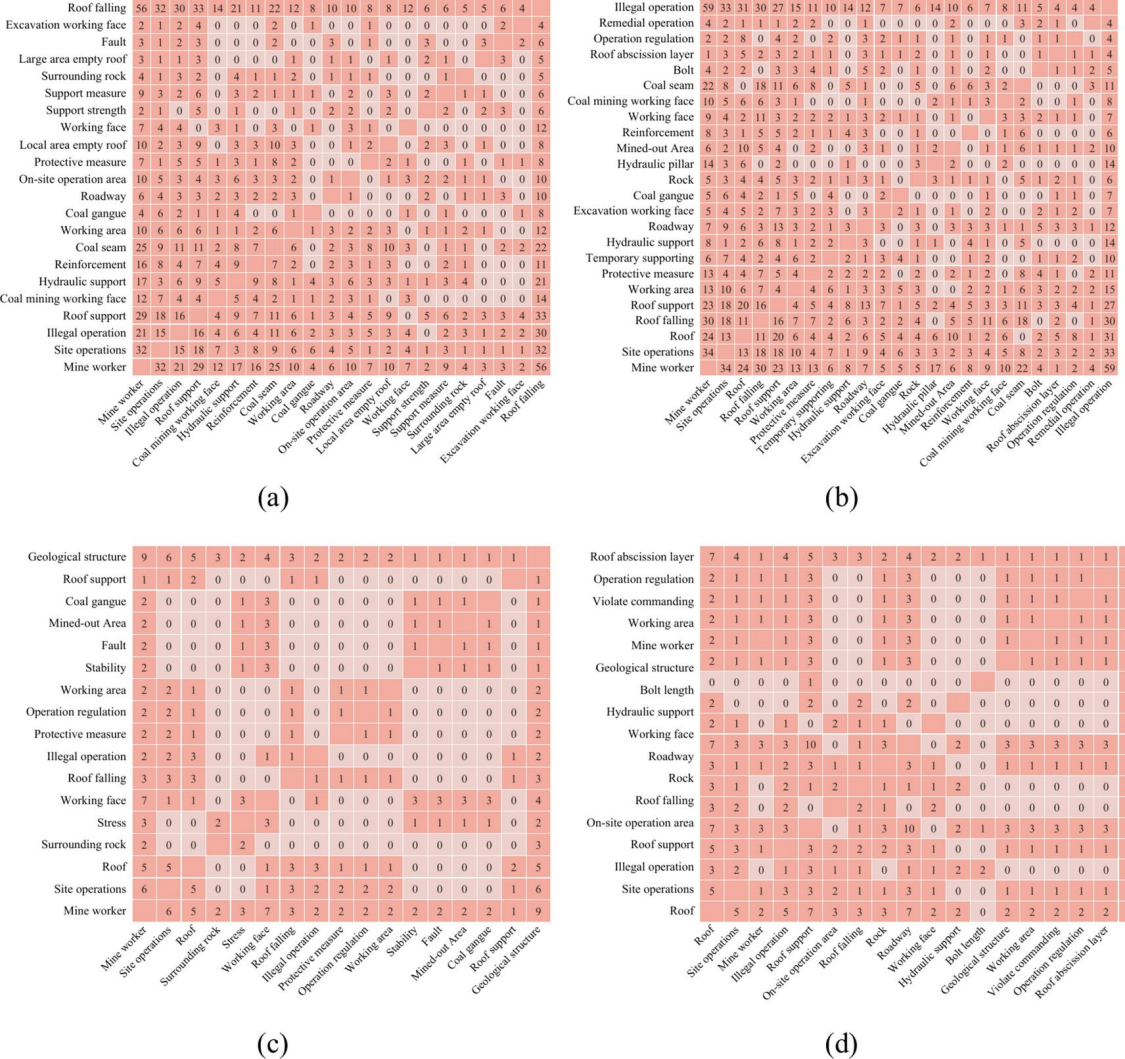
Co-occurrence matrix of hazard factors of roof accident direct causes. (**a**) Co-occurrence matrix of the ‘Roof Falling’ hazard factor; (**b**) Co-occurrence matrix of the ‘Illegal operation’ hazard factor; (**c**) Co-occurrence matrix of the ‘Geological structure’ hazard factor; (**d**) Co-occurrence matrix of the ‘Roof abscission layer’ hazard factor.

**Fig. 3:**
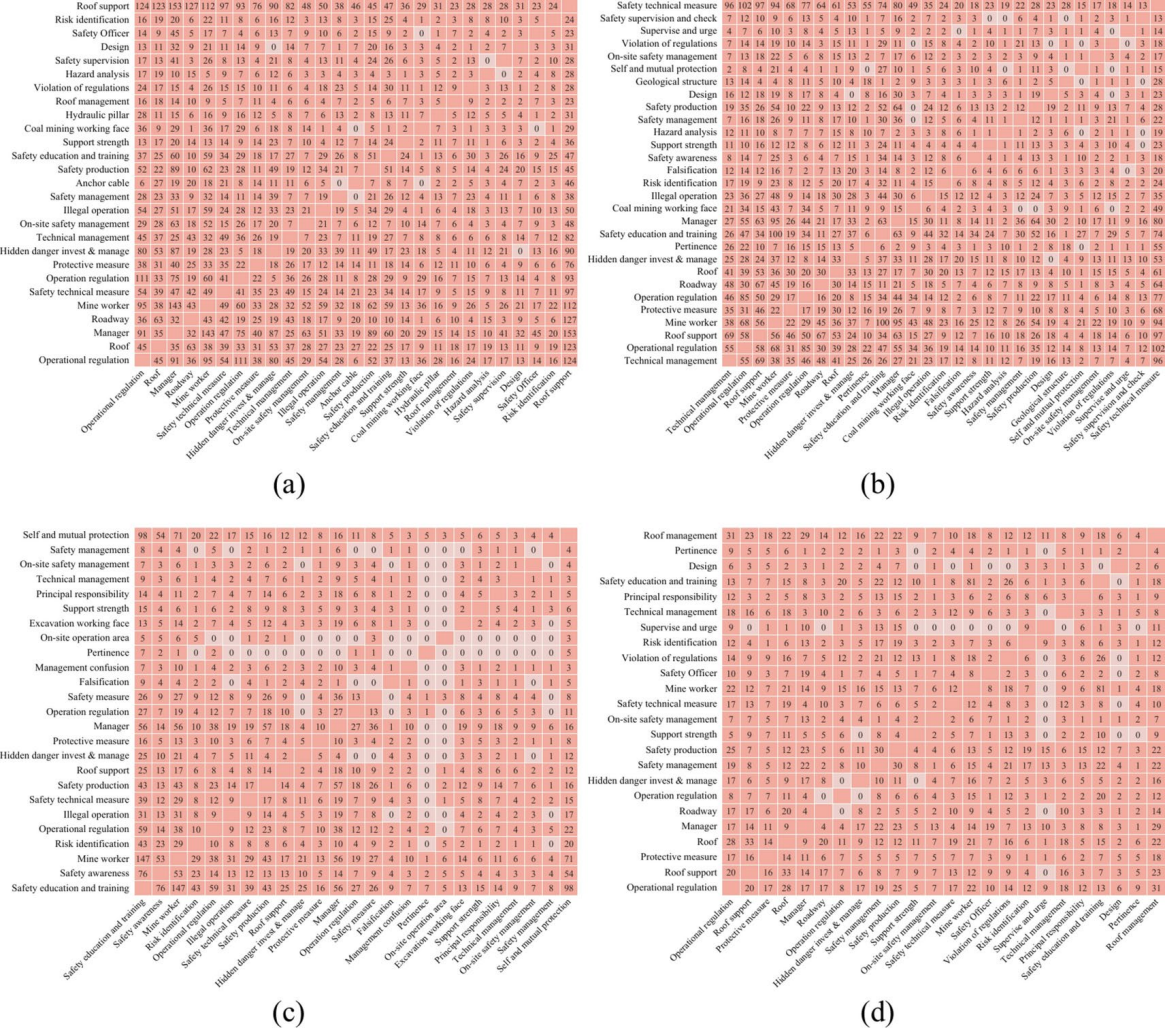
Co-occurrence matrix of hazard factors of roof accident indirect causes. (**a**) Co-occurrence matrix of the ‘Roof support’ hazard factor; (**b**) Co-occurrence matrix of the ‘Safety technical measure’ hazard factor; (**c**) Co-occurrence matrix of the ‘Self and mutual protection’ hazard factor; (**d**) Co-occurrence matrix of the ‘Roof management’ hazard factor.

Where 3/5 denotes the roof accident hazard factor of A appearing three out of five times on B’s left-side, while 2/5 is A appearing two out of five times on B’s right-side, i.e., *E*(*B*_*right*_) = *E*(*B*_*left*_). And if the combinations include three times A, B, and C hazard factors and two times D, B, and C, the left-information entropy of the hazard factor B is unchanged, but the right-information entropy is *E*(*B*_right_) = $- 1\log 1$ = 0. Here is the partial co-occurrence matrix of the hazard factor, as shown in Figs. 2 and 3, which denotes, from (a) to (d), a co-occurrence matrix of roof hazard factors, and its information entropy summarized in Tables 1 and 2.

**Table 1:** Information entropy of hazard factors of roof accident direct causes.

Series number	Hazard factor	Left-side	Right-side	Series number	Hazard factor	Left-side	Right-side
*X*_1_	Hydraulic support	3.165672	3.059489	*X*_22_	Violate commanding	1.791759	1.560710
*X*_2_	Roof support	3.337589	3.291584	*X*_23_	Support failure	1.791759	1.560710
*X*_3_	Site operations	3.317950	3.276070	*X*_24_	Bolt	2.043192	1.676988
*X*_4_	Roadway	3.062529	2.865703	*X*_25_	Mined-out Area	2.163956	2.302585
*X*_5_	Geological structure	1.560710	1.747868	*X*_26_	Fault	2.163956	2.302585
*X*_6_	Wall tapping and roof sounding	1.475076	1.475076	*X*_27_	Anchor cable	2.253858	2.484907
*X*_7_	Roof	3.536060	3.187996	*X*_28_	Stress	2.484907	2.253858
*X*_8_	Broken roof	1.791759	1.560710	*X*_29_	Working face	2.271869	2.253858
*X*_9_	Coal mining working face	1.667462	2.772589	*X*_30_	Reinforcement	1.979205	1.907284
*X*_10_	Temporary supporting	2.187322	2.394700	*X*_31_	Rock	2.057898	2.204785
*X*_11_	Operation regulation	2.079442	1.494175	*X*_32_	Protective measure	2.150060	2.278303
*X*_12_	Excavation working face	2.197225	2.484907	*X*_33_	Coal seam	2.707270	2.505290
*X*_13_	Support measure	2.965016	2.752192	*X*_34_	Working area	1.791759	1.995922
*X*_14_	Roof falling	3.613366	3.613366	*X*_35_	Roof structure	0.950271	0.950271
*X*_15_	Illegal operation	2.586588	3.465520	*X*_36_	Bolt length	0.562335	1.386294
*X*_16_	Coal gangue	2.180946	1.945935	*X*_37_	Dip angle of coal seam	1.386294	1.386294
*X*_17_	On-site operation area	1.609438	1.791759	*X*_38_	Stability	1.332179	1.609438
*X*_18_	Support strength	2.098274	1.972247	*X*_39_	Large area empty roof	1.560710	0.867563
*X*_19_	Mine worker	3.289393	3.077627	*X*_40_	Surrounding rock	1.747868	1.549826
*X*_20_	Remedial operation	1.560710	2.145842	*X*_41_	Roof abscission layer	1.945910	1.747868
*X*_21_	Hydraulic pillar	2.652588	2.890372	*X*_42_	Local area empty roof	1.945910	1.747868

**Table 2:** Information entropy of hazard factors of roof accident indirect causes.

Series number	Hazard factor	Left-side	Right-side	Series number	Hazard factor	Left-side	Right-side
*X*_1_	Manager	3.733289	3.847200	*X*_25_	Operation regulation	3.693247	3.288101
*X*_2_	Operational regulation	4.165188	4.398839	*X*_26_	Illegal operation	3.287757	3.642496
*X*_3_	Roof support	4.325126	4.458626	*X*_27_	Technical management	3.076234	3.738822
*X*_4_	Safety supervision	4.167961	4.078538	*X*_28_	On-site safety management	3.184635	3.544624
*X*_5_	Roof	4.233084	4.153253	*X*_29_	Risk identification	3.290001	3.255810
*X*_6_	Protective measure	4.006353	4.177320	*X*_30_	Roadway	4.145658	3.770995
*X*_7_	Hidden danger investigation and management	4.081025	4.200958	*X*_31_	Pertinence	2.955468	2.999272
*X*_8_	Safety production	4.304798	3.750515	*X*_32_	Violation of regulations	3.259785	3.610195
*X*_9_	Safety education and training	3.294448	3.529023	*X*_33_	Hazard analysis	3.414726	3.161017
*X*_10_	Safety management	3.874486	3.958255	*X*_34_	Hydraulic support	3.528906	3.776539
*X*_11_	Mine worker	4.083642	3.950654	*X*_35_	Safety awareness	1.703917	3.258829
*X*_12_	Safety technical measure	4.094006	4.019701	*X*_36_	Responsibility system	2.424711	3.657935
*X*_13_	Remedial operation	2.890372	2.639341	*X*_37_	Supervise and urge	3.710782	3.279449
*X*_14_	Safety measure	2.920687	2.714452	*X*_38_	Management confusion	1.906155	2.458311
*X*_15_	On-site operation area	2.811419	2.867440	*X*_39_	Mining layout	2.433699	2.579625
*X*_16_	Excavation working face	3.173103	2.755382	*X*_40_	Setting the institutions	2.798513	2.725550
*X*_17_	Roof management	3.052521	2.956494	*X*_41_	Fault	2.776913	3.152707
*X*_18_	Support strength	3.186589	2.946397	*X*_42_	Emergency disposal	2.694154	2.765587
*X*_19_	Geological structure	3.381964	3.302748	*X*_43_	Hydraulic pillar	3.282951	3.174400
*X*_20_	Principal responsibility	1.823170	2.972846	*X*_44_	falsification	2.498590	3.059489
*X*_21_	Coal mining working face	3.622898	3.387234	*X*_45_	Safety officer	2.794740	2.954003
*X*_22_	Safety supervision and check	3.550301	3.548576	*X*_46_	Anchor cable	2.989862	3.267852
*X*_23_	Safety inspection	3.451697	3.431809	*X*_47_	Design	3.289981	3.387906
*X*_24_	Self and mutual protection	2.425693	1.250501				

In each subgraph of Figs. 2 and 3, the corresponding risk factors are represented by the horizontal and vertical axes based on a specific identified hazard factor, and the values at the intersection indicate the simultaneous occurrence times in the same accident report. If the value in the co-occurrence matrix is zero, it means that the corresponding hazard factor has no co-occurrence times.

The left-side and right-side of Tables 1 and 2 are abbreviations for the left-side and right-side information entropy of each hazard factor, respectively. Moreover, the “X24 Bolt” and “X36 Bolt Length” in Table 1 seem to be similar. However, the original roof accident report description is as follows: Direct cause of the accident. On July 10 at 8 p.m., roadway repair in shift 1102N intake airflow roadway violated the regulations of “1102N working face roadway repair safety technical measures”. The workers were asked by the shift supervisor to saw *the bolts* short, and the repair area’s *bolt length* did not meet the requirements, resulting in insufficient supporting strength and a side-falling accident.

To get around this problem, the co-occurrence matrix was made by converting the hazards phrases that occurred simultaneously in the same accident report into co-occurrence values. Then calculate the corresponding information entropy using the co-occurrence matrix. This will allow for the thorough and in-depth analysis of the hidden relationship between them as well as the revelation of ontology-based semantic logic and connection. The results are as follows.

#### Identified surrogate model of sensitivity analysis

Given that there is no obvious functional equation between the hazard factors of roof accidents, if an objective function is fitted, it affects the sensitivity analysis and even the accuracy of the results. To combat this tricky problem, we need to build a surrogate model without an objective function. Common surrogate models include radial basis function models (RBF)^42^, Kriging models^42^, support vector machine regression models (SVM)^50^, neural network models^51^ etc.

Moreover, the LIBSVM surrogate model^52,53^ was developed and designed by Professor Lin Chih-Jen of the Chinese National Taiwan University for the classification, regression, and distribution estimation of SVMs. It provides an approach to handling nonlinear problems through kernel tricks and provides cross-validation functions for model selection and hyper-parameter optimization. Besides, the BP surrogate model^51^ refers to the neural network model based on the error backpropagation algorithm, which is used to approximate and predict the complex system by adjusting the weights and biases in the network in order to learn the mapping relationship between the inputs and the outputs.

This paper selects three indicators, as shown in Eqs. (21) to (23), namely the correlation coefficient (*R*^2^), the mean absolute error (MAE), and the mean bias error (MBE), as the evaluation of the surrogate model, considering that using the MBE is not the RMSE due to the fact that there may be outliers in the input data and without taking the error’s direction into account, which is to objectively analyze the model accuracy, parameter training effect, and model stability between LIBSVM and BP.

21 $$R^{2} = 1 - \frac{{SS_{res} }}{{SS_{tot} }} = 1 - \frac{{\sum\nolimits_{i}^{m} {\left( {Y_{i}^{R} - Y_{i}^{P} } \right)^{2} } }}{{\sum\nolimits_{i}^{m} {\left( {Y_{i}^{R} - \overline{Y}} \right)^{2} } }}$$

22 $$MAE = \frac{1}{n}\sum\limits_{i = 1}^{m} {\left| {Y_{i}^{R} - Y_{i}^{P} } \right|}$$

23 $$MBE = \frac{1}{n}\sum\limits_{i = 1}^{m} {\left( {Y_{i}^{R} - Y_{i}^{P} } \right)}$$

where *SS*_*res*_ denotes the residual sum of squares, i.e., the sum of squares of the differences between the actual and predicted values of the model, and *SS*_*tot*_ is the total sum of squares, i.e., the sum of squares of the differences between the actual and predicted average values of the model; *m* is the total number of samples; $Y_{i}^{R}$ represents the actual value of the *i*th variable; $Y_{i}^{P}$ is the predicted value (i.e., the test value) of the *i*th variable; and $\overline{Y}$ denotes the sample mean value.

**Fig. 4:**
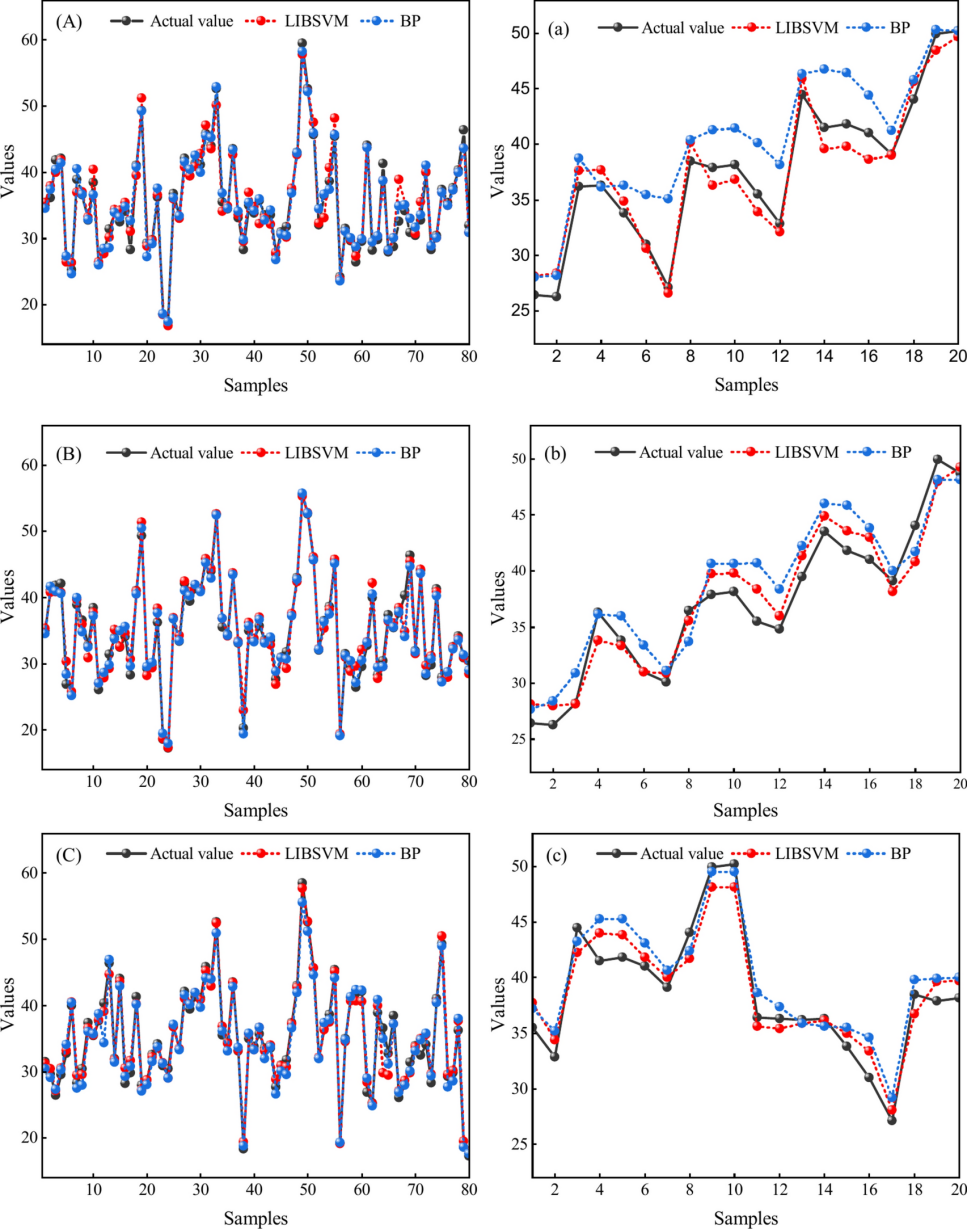

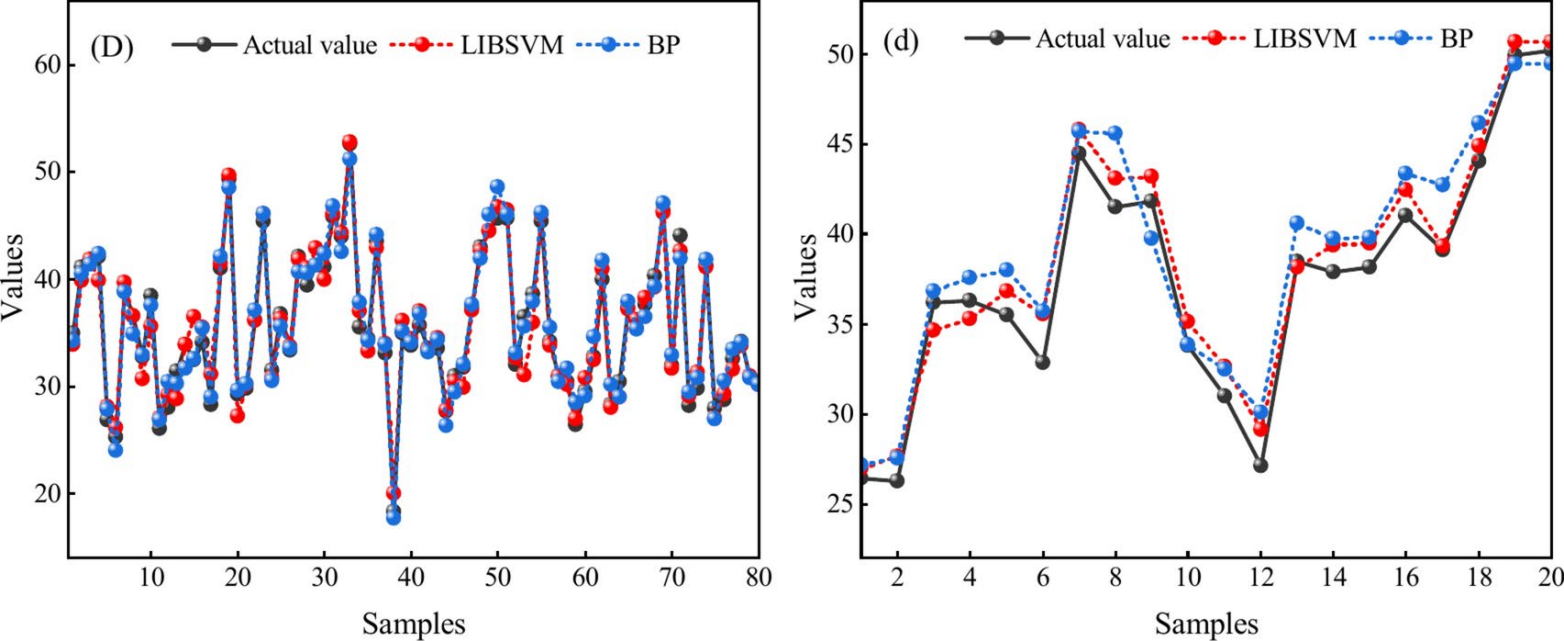
Surrogate model effects of training and testing samples; (**A**) Training samples-1; (**a**) Testing samples-1; (**B**) Training samples-2; (**b**) Testing samples-2; (**C**) Training samples-3; (**c**) Testing samples-3; (**D**) Training samples-4; (**d**) Testing samples-4;

In order to maintain a 4 to 1 ratio between the training and test groups—that is, the first 80 sets of data are used as the training dataset and the rest of the 20 sets are used as the test dataset—100 sets of data with an error range of plus or minus 15% are randomly generated using the information entropy of each hazard factor as a benchmark. Then, using the MATLAB software, Fig. 4 illustrates the training and testing effects between the LIVSVM and the BP surrogate model, respectively. The capital letters A through D stand for the training samples, and the corresponding lowercase letters represent the testing samples.

As summarized in Tables 3 and 4, it then obtained the *R*^2^, MAE, and MAB results of the surrogate model after finishing the multiple tests. The further details are as follows.

**Table 3:** Statistical results for the surrogate model testing.

Surrogate model	Series number	Training samples			Testing samples		
		*R*^2^	MAE	MBE	*R*^2^	MAE	MBE
BP	1	0.97443	1.84434	− 0.05331	0.81578	3.11009	− 2.45443
	2	0.95406	1.39660	0.19798	0.84766	3.04011	− 2.66547
	3	0.95697	0.94554	− 0.07334	0.86578	2.91676	0.81676
	4	0.96587	1.17887	− 0.43899	0.85341	3.09301	− 1.45619
LIBSVM	1	0.94369	1.32558	0.10440	0.89736	2.73582	1.18731
	2	0.96667	0.94879	− 0.07332	0.90752	2.74706	− 0.83476
	3	0.97708	0.91258	0.12897	0.93602	2.38605	0.16236
	4	0.96544	1.12227	− 0.08315	0.91354	2.67384	− 1.03496

**Table 4:** Statistical analysis of the surrogate model.

Surrogate model	Training samples			Testing samples		
	*R*^2^	MAE	MBE	*R*^2^	MAE	MBE
BP	0.96283	1.34134	− 0.09192	0.84566	3.03999	− 1.43983
LIBSVM	0.96322	1.07731	0.01923	0.91361	2.63569	− 0.13001

Table 4 shows the average values of each indicator, illustrating that the LIBSVM surrogate model should be selected and identified as the analysis tool of the global sensitivity, firstly because, although the training and the testing samples are quite similar in terms of the *R*^2^, the LIBSVM shows marginally better; moreover, the MAE value of the LIBSVM is smaller than the BP in both the training and the testing samples; and lastly, the LIBSVM’s MBE value is closer to zero, fully indicating that there is no obvious systematic bias and the model is more accurate and stable.

#### Stability design of sample sampling algorithm

The design of the sample sampling algorithm is, without a doubt, a significant step in Sobol’s sensitivity analysis method for roof hazard factors because the proper sample size will affect the stability and accuracy of the 1th and the global sensitivity results. Some^54^ believed that Latin hypercube sampling employs stratified sampling, where the interval of each variable value is divided into *N* segments (commonly *N* is equal to the number of samples in an M*N hypercube, where *M* is the dimensionality of the input variable), and pseudo-random numbers are generated in each of the selected interval segments to ensure that each sample point has different coordinates in order to overcome the drawbacks of the Monte Carlo method.

**Fig. 5:**
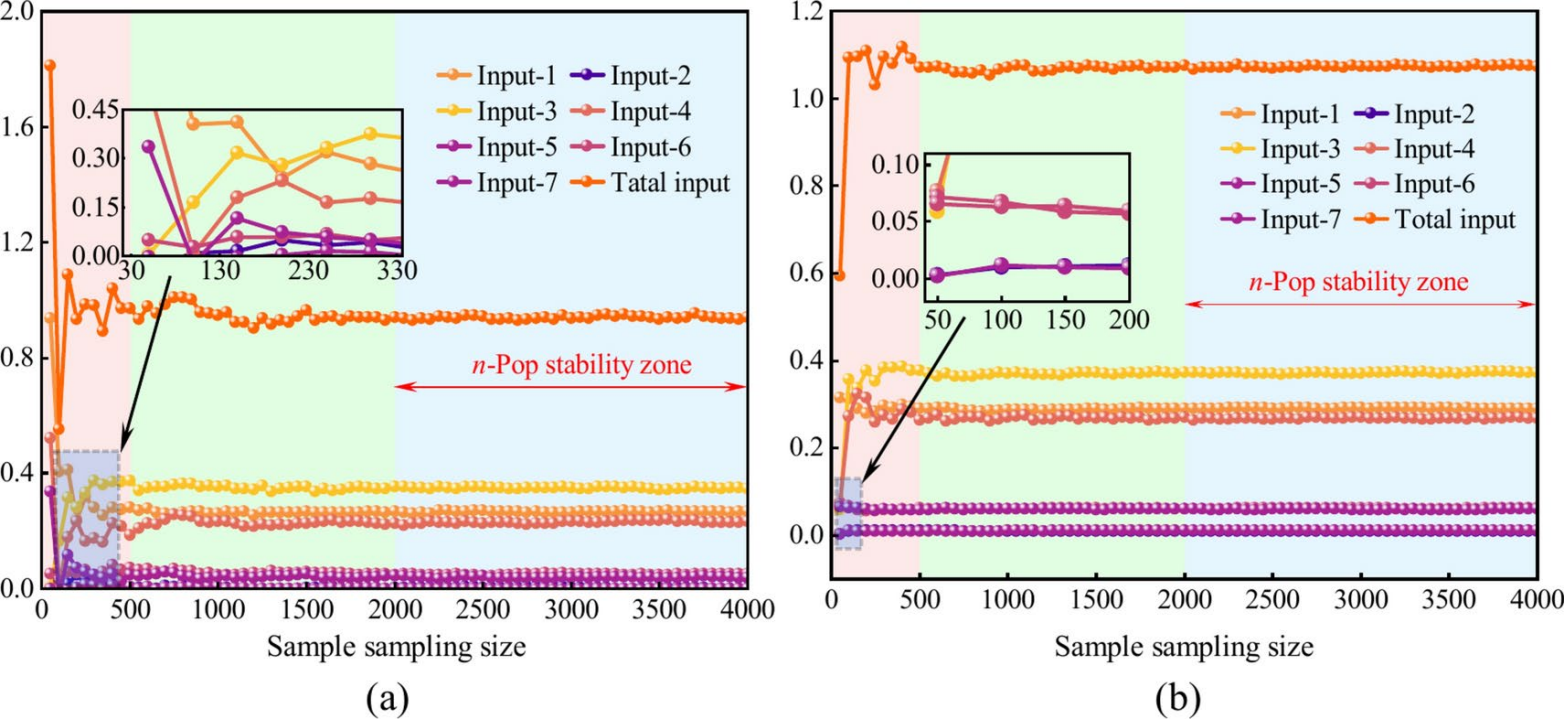
Sample sampling stability design for the roof accident hazard factors. (**a**) The 1th sensitivity sampling design; (**b**) The global sensitivity sampling design;

Considering the hazard factors, including 42 direct and 47 indirect causes, every 7 hazard factors are taken as the same initial inputs, and using Latin hypercube sampling to traverse the sampling scale based on the *n*Pop = 50:50:4000 and carry out the 1th and global sensitivity sampling size stability tests^55^, as shown in Fig. 5.

Figure 5 also shows the stability of the total input, both the first and global sensitivities of sample sampling size, indicating overall stability of the other seven variables when they are used as initial inputs. Obviously, the sensitivity results of each hazard factor generally converge and tend to be stable and no longer fluctuate with the increase of the sampling size once it reaches 2000 (i.e., the light blue color area in Fig. 5, namely the *n*Pop stability zone, and can ensure effectively the accuracy of the sensitivity results.

### Result analysis

**Fig. 6:**
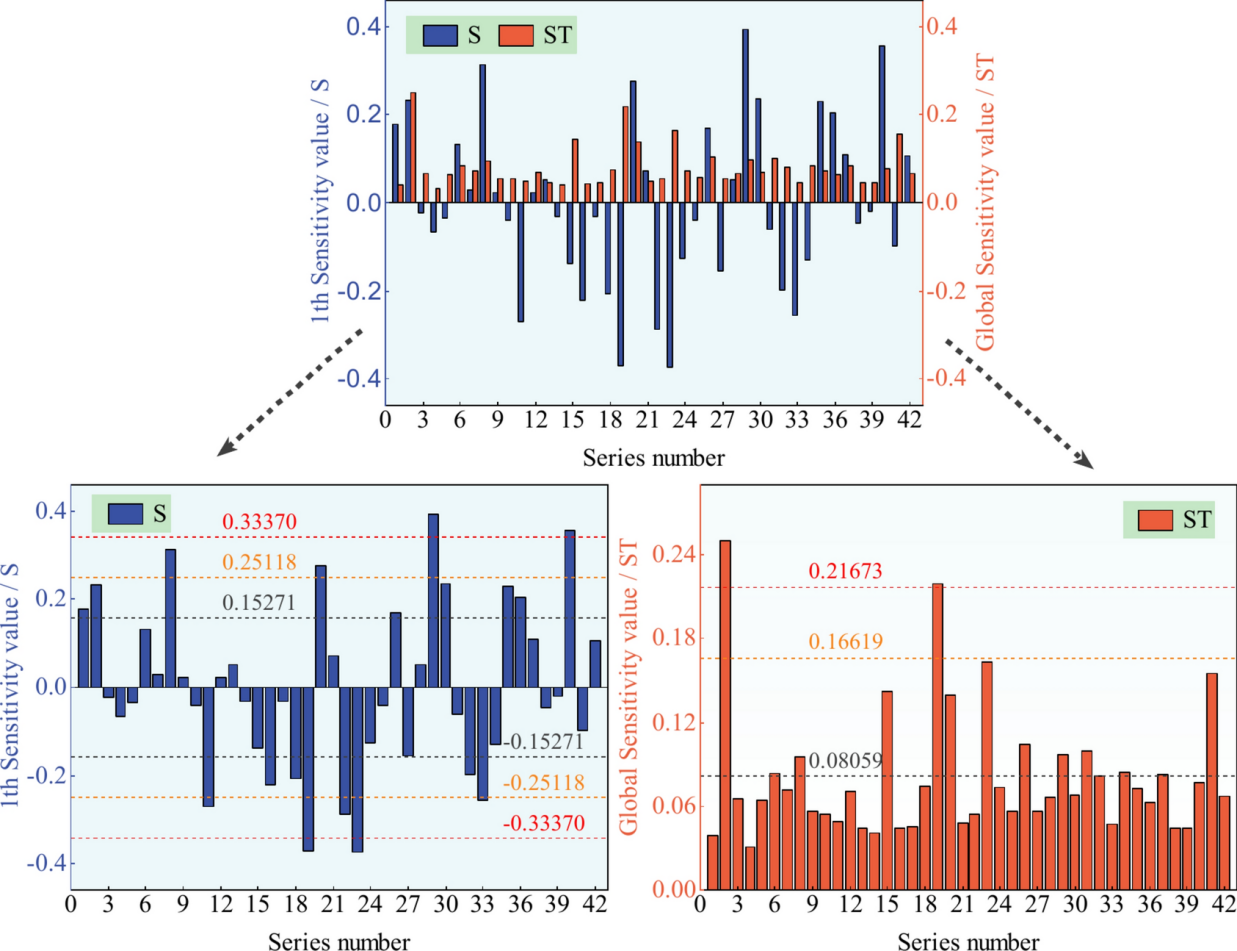
Sensitivity results of hazard factors in roof accidents’ direct causes.

**Fig. 7:**
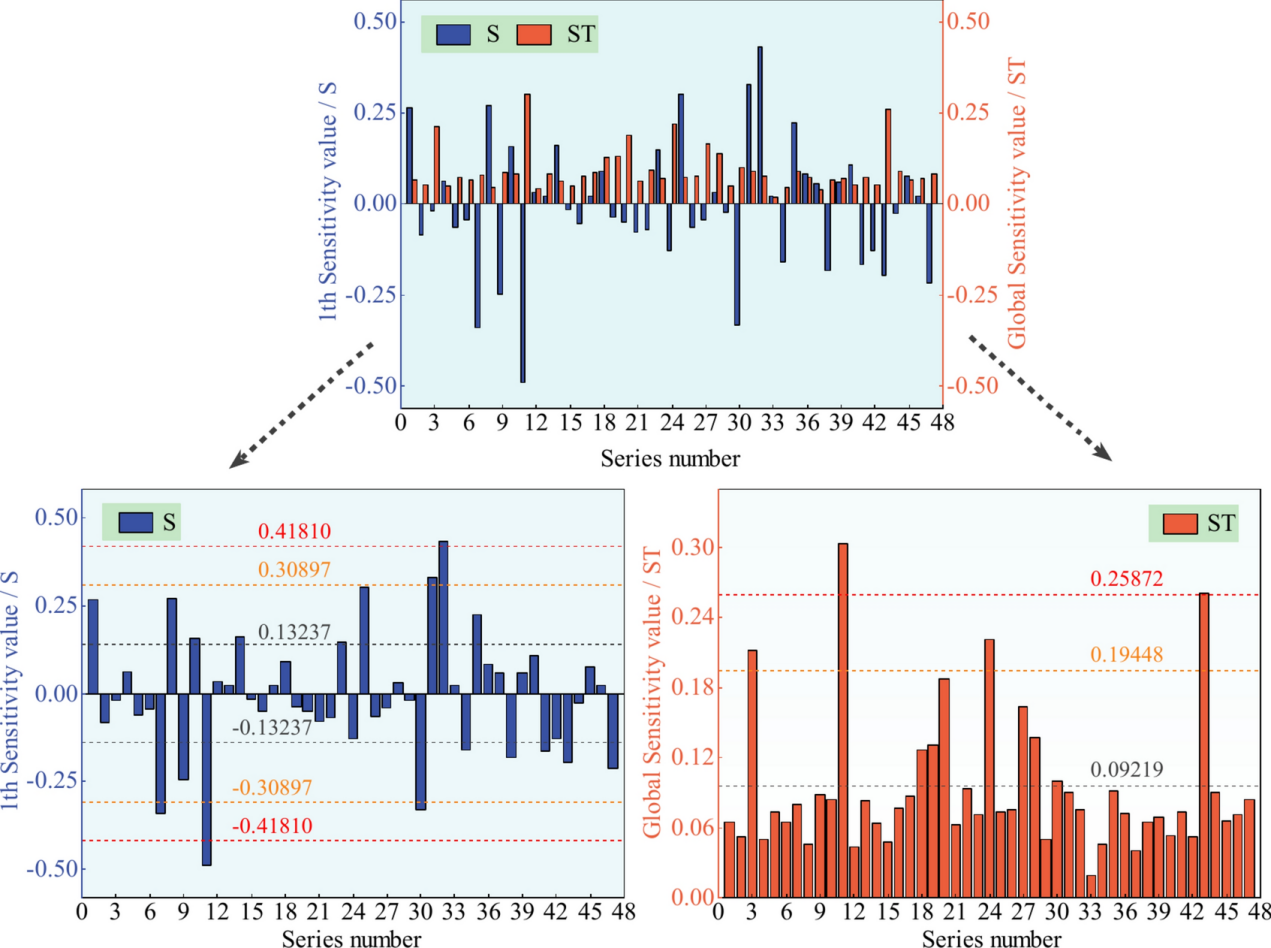
Sensitivity results of hazard factors in roof accidents’ indirect causes.

Considering all the preprocessing steps, set *n*Pop = 2000, chose the LIBSVM surrogate model, established the upper and lower boundaries of hazard factors’ information entropy, and adopted the own function Sobolset to improve the sample richness. Then, Sobol’s method was used to obtain the results of roof accident hazard factors, respectively, as pictured in Figs. 6 and 7.

Each picture in Figs. 6 and 7 includes two parts: on the top is the full view, and then it is divided into two segments: the left denotes the 1th sensitivity expressed with S (i.e., each input factor is independent and disregards their interaction), and the right part is the global sensitivity signed with ST (i.e., each input factor needs to consider their interaction).

As shown in Figs. 6 and 7, the mean value, the golden section value^56^, and the secondary golden section value (i.e., the value that is calculated by combining the golden section value with the maximum value once more) are represented, respectively, by three groups of symmetrical dotted lines in the left S-image and three dotted lines in the right ST-image, which are dark grey, orange, and red from the 0-axis to the far.

Sort the hazard factors into four levels, and then use the series number from Tables 1 and 2 and the sensitivity results from Figs. 6 and 7 to summarize in Tables 5 and 6, respectively, and display the top three levels; the rest below the average value are not shown. Note: The minus sign in the S value denotes that this input change direction is opposite to the output, having the opposite effect.

**Table 5:** Sensitivity statistics of hazard factors in roof accidents’ direct causes.

Type	Rank	Series number	Hazard factor	S	Rank	Series number	Hazard factor	ST
I	1	*X*_29_	Working face	0.39386	1	*X*_2_	Roof support	0.24973
	2	*X*_23_	Support failure	− 0.37488	2	*X*_19_	Mine worker	0.21887
	3	*X*_19_	Mine worker	− 0.37040				
	4	*X*_40_	Surrounding rock	0.35564				
II	5	*X*_8_	Broken roof	0.31218				
	6	*X*_22_	Violate commanding	− 0.28689				
	7	*X*_20_	Remedial operation	0.27673				
	8	*X*_11_	Operation regulation	− 0.27121				
	9	*X*_33_	Coal seam	− 0.25662				
III	10	*X*_30_	Reinforcement	0.23636	3	*X*_23_	Support failure	0.16334
	11	*X*_2_	Roof support	0.23338	4	*X*_41_	Roof abscission layer	0.15482
	12	*X*_35_	Roof structure	0.23067	5	*X*_15_	Illegal operation	0.14234
	13	*X*_16_	Coal gangue	− 0.22081	6	*X*_20_	Remedial operation	0.13929
	14	*X*_18_	Support strength	− 0.20757	7	*X*_26_	Fault	0.10458
	15	*X*_36_	Bolt length	0.20332	8	*X*_31_	Rock	0.09970
	16	*X*_32_	Protective measure	− 0.19739	9	*X*_29_	Working face	0.09692
	17	*X*_1_	Hydraulic support	0.17868	10	*X*_8_	Broken roof	0.09542
	18	*X*_26_	Fault	0.16996	11	*X*_34_	Working area	0.08383
	19	*X*_27_	Anchor cable	− 0.15589	12	*X*_6_	Wall tapping and roof sounding	0.08381
					13	*X*_37_	Dip angle of coal seam	0.08229
					14	*X*_32_	Protective measure	0.08165

**Table 6:** Sensitivity statistics of hazard factors in roof accidents’ indirect causes.

Type	Rank	Series number	Hazard factor	S	Rank	Series number	Hazard factor	ST
I	1	*X*_11_	Mine worker	− 0.48944	1	*X*_11_	Mine worker	0.30271
	2	*X*_32_	Violation of regulations	0.43050	2	*X*_43_	Hydraulic pillar	0.26039
II	3	*X*_7_	Hidden danger investigation and management	− 0.34041	3	*X*_24_	Self and mutual protection	0.22111
	4	*X*_30_	Roadway	− 0.33134	4	*X*_3_	Roof support	0.21190
	5	*X*_31_	Pertinence	0.32837				
III	6	*X*_25_	Operation regulation	0.30269	5	*X*_20_	Principal responsibility	0.18757
	7	*X*_8_	Safety production	0.26896	6	*X*_27_	Technical management	0.16432
	8	*X*_1_	Manager	0.26550	7	*X*_28_	On-site safety management	0.13770
	9	*X*_9_	Safety education and training	− 0.24624	8	*X*_19_	Geological structure	0.13080
	10	*X*_35_	Safety awareness	0.22337	9	*X*_18_	Support strength	0.12625
	11	*X*_47_	Design	− 0.21501	10	*X*_30_	Roadway	0.09969
	12	*X*_43_	Hydraulic pillar	− 0.19724	11	*X*_22_	Safety supervision and check	0.09330
	13	*X*_38_	Management confusion	− 0.18268				
	14	*X*_41_	Fault	− 0.16398				
	15	*X*_14_	Safety measure	0.16075				
	16	*X*_34_	Hydraulic support	− 0.15969				
	17	*X*_10_	Safety management	0.15685				
	18	*X*_23_	Safety inspection	0.14657				

According to the results of Tables 5 and 6, hazard factors have been divided into types I, II, III, and IV based on their sensitivity values, corresponding to priority management, priority prevention, general management, and general prevention, respectively. And it was found in Table 5 that, disregarding the interactions between factors (i.e., the 1th sensitivity value, S), for the direct cause of roof accidents, there were four hazard factors in type I, accounting for a total of 9.52%, and five risk factors in type II, occupying 11.90%; the rest totally occupies 78.58%. While considering the interactions (i.e., the global sensitivity value, ST), only two hazard factors belong to type I and nothing to type II.

Similarly, in Table 6 for the indirect cause, it can be concluded that, disregarding the interactions between factors (i.e., value-S), there were two hazard factors in type I, accounting for a total of 4.26%, and three risk factors in type II, occupying 6.38%; the rest totally occupies 89.36%. While considering the interactions (i.e., value-ST), types I and II have only two hazard factors, respectively.

### Discussion

Considering that the approach must be reasonable and the hazard factors as the initial input must be accurate. In addition, the number of accident reports will continue to rise in the future as an inexorable trend, and coal mining remains a large and complex system. The superimposed risk or coupling effect can be obtained by oversimplifying the model and forgetting whether these risk factors belong to the same accident report. It is difficult to imagine obtaining the results based on simple arithmetic operations or prior and posterior probability. Here is a brief explanation of some potential disadvantages of the currently used methods that are mentioned in the introduction:

(1) Decision tree framework. Although it does well on classification problems, it may not be able to handle continuous variables or complex variable interactions. (2) Fault tree analysis. The primary function of fault tree analysis is to identify individual factors within a system that could cause failure; however, it might not be sufficient to capture nonlinear relationships between factors. (3) Monte Carlo method. The Monte Carlo method uses random sampling, and the computational cost is high. The accuracy of the results is also limited by the sample size and sampling quality. (4) Weibull and Poisson distribution models. These statistical models usually assume that the data follow specific distributions, but in practice, accident data might not precisely match these distributions, which could affect the model’s predictions. (5) Time series theory analysis. Time series analysis may not sufficiently account for interactions among several factors because it usually focuses on changes in a single variable over time. (6) Fuzzy comprehensive evaluation method. Although fuzzy logic can deal with uncertainty, it might not be accurate enough for quantitative analysis of complex factor interactions. (7) Bayesian networks. Conditional dependencies between variables can be handled by Bayesian networks; however, they are sensitive to incorrect network structure specification and require expert knowledge to build. (8) Triangular fuzzy theory and mutation theory. These approaches have some advantages when handling ambiguity and uncertainty, but in practical applications, they may need large amounts of parameter adjustment and subjective assessment. (9) DEMATEL-ANP methods and complex networks. Although these methods are useful in identifying and analyzing factors and their relationships in complex systems, they have some limitations in handling large-scale data.

Compared to other papers, the research object in this paper and other similar studies was reports of coal mine roof accidents. However, in contrast to the current methodology and approach, we tried the following new approach: after using optical character recognition technology to convert these accident reports into text format of UTF-8 or GBK, we were able to obtain hazard factors after using Hanzi Regular and Chinese word segmentation to cut them into individual words (phrases) that represent the key information details of the roof accidents’ causes and to retain high-frequency occurrences that correspond with the accidents.

Then the co-occurrence matrix was created by converting the hazards phrases that occurred simultaneously in the same accident report into co-occurrence values and allocating the initial input values of the hazards. Moreover, intelligent algorithms of the surrogate model with appropriate classification prediction performance can be used to obtain a better function mapping relationship. In this processing, the algorithm could imitate and perceive the potential relation by using some of the hazards as training samples and the remaining as testing samples. Lastly, construct a calculation process that uses multiple effect functions to solve hazards interaction effects by using Sobol’s method.

It may or may not be accurate to build the inner relationship network diagram using prior knowledge and cognition of coal mine accidents. Assume you are a newbie in this field. You will miss the opportunity to explore the true interaction between hazard factors if you try to solve this Gordian knot without breaking out of stereotypes. Theoretically, if our theory and methodology are correct more and more, it seems that this method can be applied and extended to other types of coal mine accident analysis; however, more accurate and reasonable processing approaches should be investigated, as should improvements to the methods of assigning initial input values and to the text mining hazards factors.

## Conclusion

To better draw lessons from prior reports of coal mine roof accidents and prevent them before they occur, this paper provides a novel methodology—obtain the hazard factor sensitivity while considering the multiple or single effects of risk factors, whether they interact or not. The following points can be concluded:

1. For the direct causes of roof accidents, when disregarding the hazard factors’ interaction, this should be priority management, e.g., working face, support operation, stability of surrounding rock, and mine worker; and the priority prevention events are broken roof, violation commanding, remedial operation, operation regulation, and coal seam. While considering the risks’ interaction, we only need priority management for two events, such as roof support and mine workers.
2. For the indirect causes, if disregarding interaction, this is priority management, e.g., mine worker and the behavior of violating regulations; and the hidden danger investigation and management, roadway stability, and the pertinence of corresponding operation regulations should be priority prevention. While considering interaction, priority management is mine worker, regular remedial and inspection hydraulic pillar; and self and mutual protection and roof support is priority prevention events.
3. By analyzing the hazard factors from 115 roof accident reports, we found that transforming the co-occurrence phrases and their co-occurrence values into a co-occurrence matrix would avoid the weakness of both text numericization and assigning the initial input values, as well as making the effect of the inner network relationship of risk elements less. No one would deny the fact that, after all, it’s much simpler to count values than to figure out complex network relationships.
4. Using the LIBSVM surrogate model, with excellent features of classification and prediction, the dilemma of the unknown objective function can be avoided; meanwhile, Sobol’s method can not only obtain the 1th sensitivity but also the global sensitivity of hazard factors, considering the inner interaction-effect relationship while avoiding its complex relationship. Overall, this method has been proposed with the aim of assessing the sensitivity of roof hazard factors objectively and comprehensively.

In conclusion, by combining the information entropy, the surrogate models, and Sobol’s method, the approach mentioned in this study may enhance the precision and effectiveness of risk assessment while offering a new tool for evaluating the coal mine roof hazard factors. It does add the possibility to evaluate the sensitivity of roof hazard factors more precisely, which is important for setting clear and prioritized management goals for coal mine safety management. However, it seems to have encountered some limitations or challenges currently, such as:

1. High-quality accident reports and model assumptions. The accuracy and effectiveness of research largely depend on the quality of input data and reasonable assumptions. The accuracy of the sensitivity analysis could be impacted if the historical roof accident report contains mistakes, omissions, or missing knowledge. It is necessary to make certain reasonable assumptions about the interactions between hazards when constructing the co-occurrence matrix to numerically represent the original input variable, and whether these assumptions can fully reflect the complex interactions still needs further refinement and verification.
2. Reliability and generalizability of the algorithm model. The methodology of this study does promote the transformation of roof accident hazard assessment from empirical judgment to scientific and quantitative avoided human interference during the sensitivity processing, in contrast to other methods mentioned in the introduction that obtain the results based on simple arithmetic operations or prior and posterior probability to achieve the superimposed risk or the coupling effect, which oversimplified the model and forgot whether these hazards belong to the same accident report. More samples may be needed to ensure the stability and reliability of the results in the subsequent exploration of the sensitivity analyses and the complex network relationships of the roof hazards in coal mines, even though the LIBSVM surrogate model performed better in this study. It is still necessary to confirm whether these models can generalize to other types of coal mine accidents.
3. Application of visualization research results. It will ultimately come down to practice if these studies become mature and form a visual analysis tool that can immediately analyze an accident report or a class of reports with similar circumstances. These technologies and methods can clearly demonstrate to mining practitioners which hazards are the main causes of roof accidents, enhance their awareness and vigilance to these factors, improve their safety consciousness, and encourage them to adopt safety precautions. In safety education and training, the visualization results can serve as a foundation, which can teach miners how to prevent and control roofing hazard risks. It can also help practitioners improve their emergency response skills and safety knowledge, such as by implementing more safety measures in high-risk areas or carrying out more thorough risk assessments prior to operations. Additionally, the visualization findings give managers a scientific foundation for decisions about mining safety management and help them in allocating resources in a way that minimizes the risk of the most sensitive factors when resources are limited.

## Data Availability

Data used to support the findings of this paper will be made available on request from the corresponding author.
